# Radial nerve branches to the triceps brachii: cadaveric analysis and surgical implications for neurotization

**DOI:** 10.1007/s00276-026-03855-5

**Published:** 2026-03-24

**Authors:** Marc Mombellet, Christophe Destrieux, Ramy Samargandi, Guillaume Bacle

**Affiliations:** 1 Department of Orthopaedic Surgery, Surgery of the Hand and Peripheral Nerves, Tours University Hospital Centre, Tours Medical University, Av. De la République, François Rabelais, Tours, France; 2 Anatomy laboratory, INSERM, Imaging Brain & Neuropsychiatry iBraiN U1253, Tours university, Tours, 37032 France; 3 Department of Orthopedic Surgery, College of Medicine, University of Jeddah, Jeddah, 23218 Saudi Arabia

**Keywords:** Radial nerve, Triceps motor branches, Neurotization, Nerve transfert, Brachial plexus

## Abstract

**Purpose:**

The radial nerve, originating from C5 to C8 and occasionally T1, is one of the two major nerves in the posterior brachial plexus bundle. Studies on the radial nerve vary regarding the number, arrangement, and emergence of its branches. This study aims to clarify the motor branch layout of the radial nerve to identify the optimal branch for neurotization.

**Methods:**

Thirty cadaveric dissections were performed using a posterior approach, spliting the triceps muscle heads.

**Results:**

Four distinct motor branches were consistently identified, all emerging before the nerve contacts the radial sulcus. Only the inferior branch to the medial head showed variability, sometimes giving additional branches to the lateral head, sensory branches, or both.

**Conclusion:**

We recommend the long head branch for neurotization due to its consistent anatomical position and reliability.

**Level of evidence:**

IV

## Introduction

Traumatic injury to peripheral nerve trunks results in major functional deficits. If there is no recovery in the innervation territory of a repaired or contused peripheral nerve trunk within 6 months, neurotization should be considered [1]. This involves transferring a healthy branch to the damaged nerve near the target muscle.

Intra-plexic nerve transfer for an isolated axillary nerve deficit is usually carried out via one of the branches of the radial nerve, the results of which have proved better than other transfers [2, 3]. The branch of the long head and then the inferior medial branch of the triceps brachialis were studied for neurotization of the axillary nerve [4–6]. The functional results and morbidity described in the literature seem comparable for the transfer of these two branches [7].

Anatomical studies have shown variable results concerning the number, topography and destinations of the motor branches of the radial nerve for the triceps brachialis, as well as an inconstant participation of the axillary or ulnar nerves in the innervation of this muscle [8–15]. The aim of this study is to describe the anatomy of the motor branches of the radial nerve destined for the triceps brachialis, to identify the constant innervation branches and the anatomical variations, and to propose reliable reference points for the choice of a donor branch with a view to neurotization.

## Materials and methods

An anatomical study based on thirty post-mortem upper limb dissections was carried out on 15 fresh-frozen cadavers. The subjects were 11 Caucasian women and 4 Caucasian men. The mean age was 87 years (min-max :79–99).

The specimens came from the body donation program of the Tours Faculty of Medicine, for research purposes. Dissections were performed by a single operator following a standardised protocol in the Tours anatomy laboratory. Cadavers with scars or a history of surgery in the area of interest were excluded. The project was approved by the Scientific, Ethical and Pedagogical Committee of the Tours Faculty.

Cadaver preparation and dissection protocol.

The bodies were positioned in the lateral decubitus position, the arm resting on a block with the shoulder at 90° of antepulsion and medial rotation, the elbow bent at 90°.

The skin incision was centred on the posterior aspect of the arm, joining the posterolateral angle of the acromion to the tip of the olecranon. The radial nerve and its branches were exposed distal to the teres major tendon after dividing the muscle heads of the triceps brachialis.

Measurement of anatomical parameters.

Measurements were taken using a ruler and a calliper graduated to the half-millimetre.

**Fig. 1:**
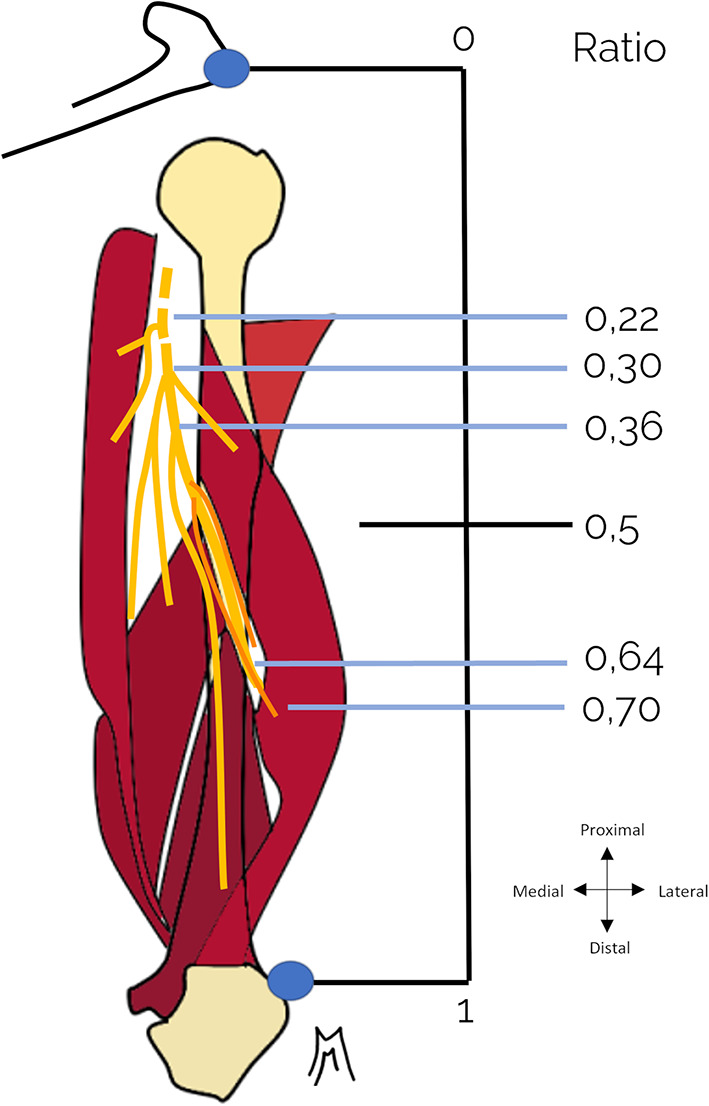
Emergence ratio of radial nerve branches at the arm, expressed as a function of the length of the patient’s arm (cm), in relation to the posterior acromial angle.

The various anatomical measurements were based on reference bony landmarks: the posterolateral angle of the acromion proximally and the distal humeral bi-epicondylar horizontal line. Measurements of the emergence point of the branches correspond to their distance from the inferior edge of the teres major. The average length of the branches is defined by the distance between their points of emergence and muscle penetration. Measurements of the origin of the nerve branches of the radial nerve were systematically related to the length of the limb, allowing it to be expressed in ratio from the posterolateral acromial angle (Fig. 1) in order to limit the influence of inter- and intra-individual differences in limb length.

### Statistical analysis

The initial assessment of a possible normal distribution of the measurements used a Shapiro statistical test. The results for measurements with a Gaussian distribution could be expressed with a 95% confidence interval (alpha risk: 0.05).

## Results

### Mapping and macroscopic aspects of the radial nerve trunk

The mean acromio-bi-epicondylar distance was 30,9 ± 2, 3 cm.

The inferior edge of the terres major tendon was 22,8 ± 1,8 cm above the bi-epicondylar line (min :19,40, max :25, ratio: 0,26).

**Fig. 2:**
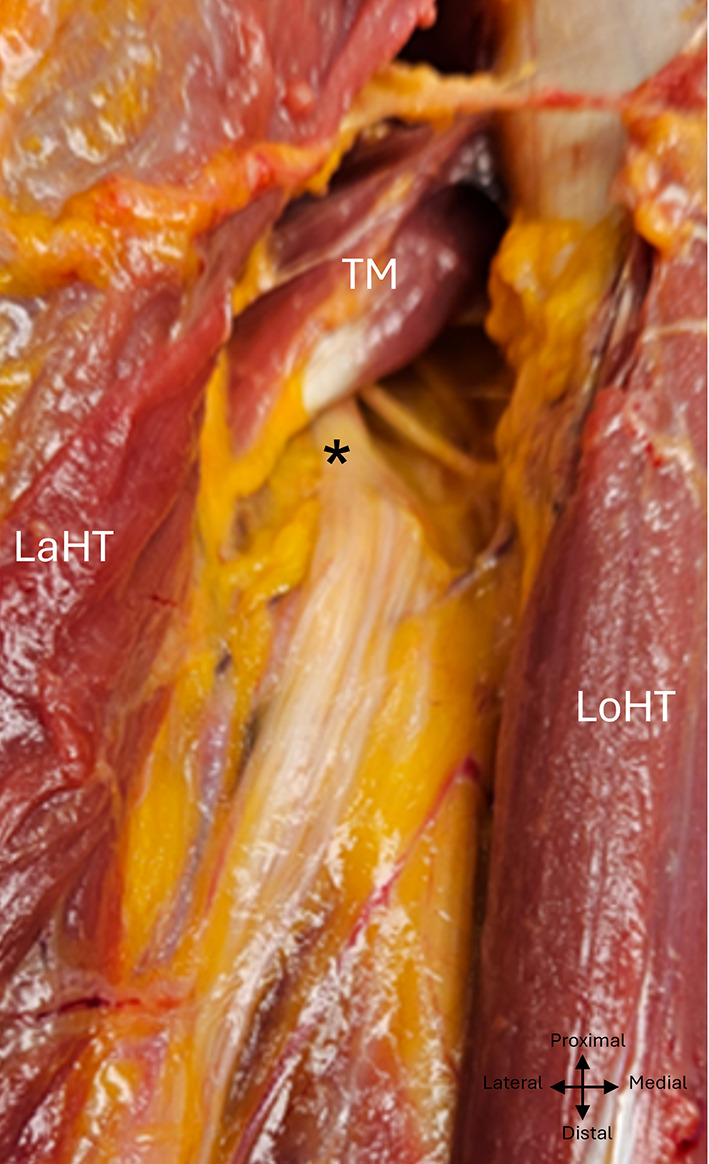
Fascicular aspect of the radial nerve in the left arm exposed posteriorly. TM, Teres major; Lath, Lateral head of triceps; Loht, Long head of triceps; asterix: radial nerve

The radial nerve was visible anterior to the tendon of the teres major muscle, at its inferior edge. It took an oblique course from proximal to distal and medial to lateral, passing through the inferior axillary space before making contact with the posterior surface of the humerus 18,8 ± 1,5 cm 1.5 cm from the bi-epicondylar line (IC à 95% (15,8–21,8), min: 16, max :21,5, ratio 0,39). The macroscopic appearance of the radial nerve corresponded to a group of nerve branches, joined together in the same epineural sheath, corresponding to the trunk of the radial nerve and its motor branches destined for the heads of the triceps brachialis as well as the two sensory branches. (Fig. 2).

**Fig. 3:**
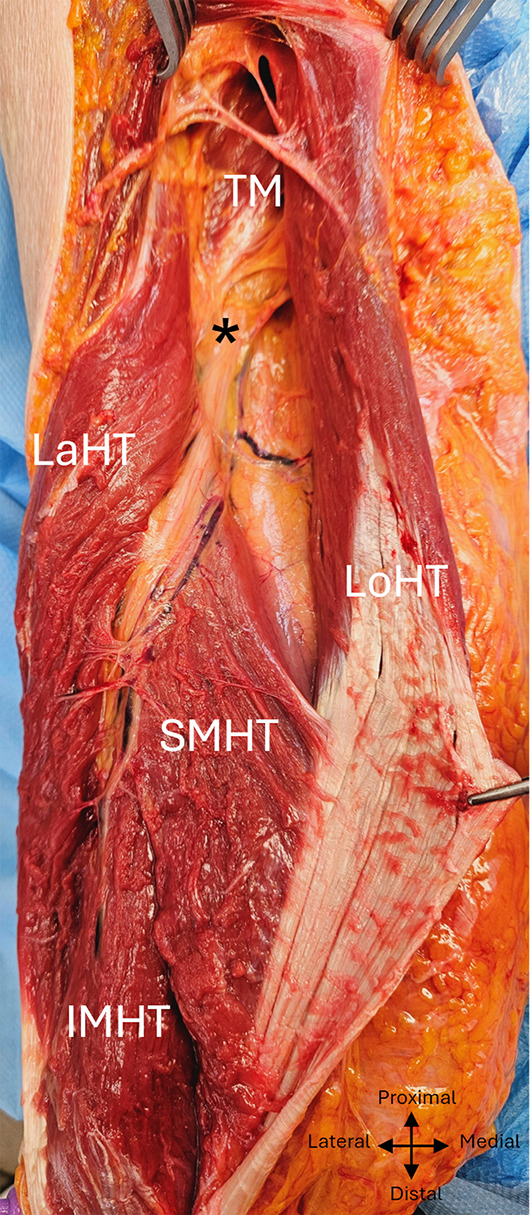
Path of the radial nerve exposed via a posterior approach to the left arm. TM, Teres major; Lath, Lateral head of triceps; Loht, Long head of triceps; SMHT, Superior medial head of triceps; IMHT, Inferior medial head of triceps; asterix: radial nerve

The radial nerve reached the anterior compartment of the arm by passing through the fibrous arch of insertion of the lateral head of the triceps brachialis, the upper and lower limits of which were situated respectively at 14,3 ± 1,1 cm (min :12,2, max :16,5) and 11,2 ± 0,9 cm (min :9,5, max :13, ratio : 0,64), from the bi-epicondylar line. (Fig. 3).

**Fig. 4:**
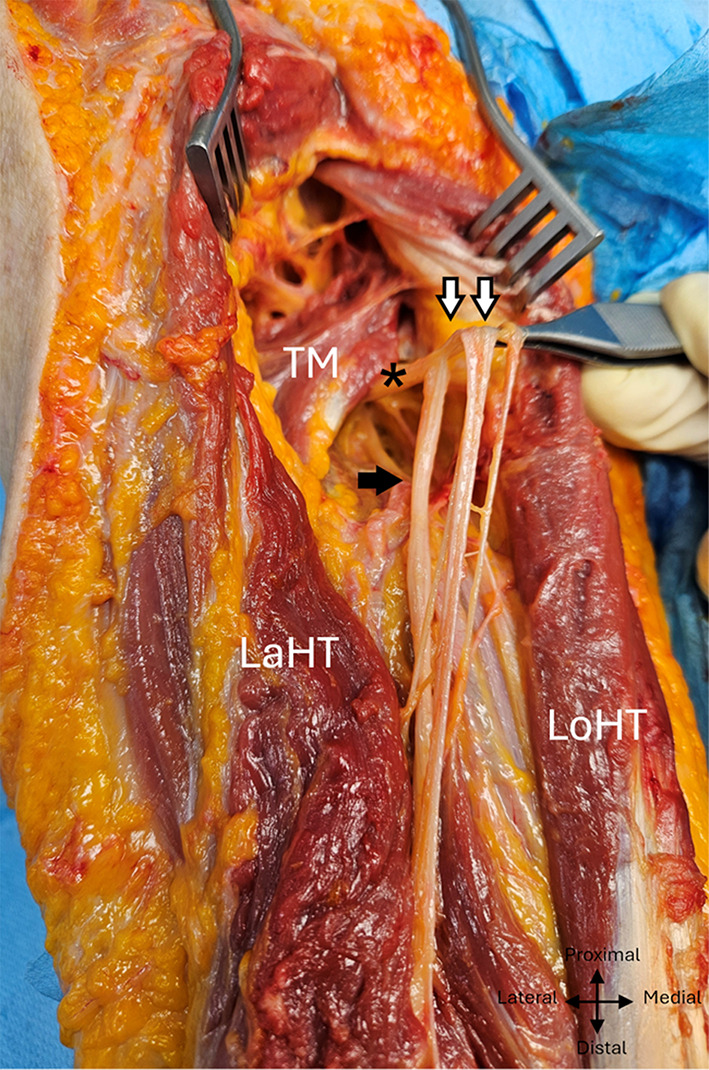
Common high division of the branches of the radial nerve in the left arm into a posteromedial bundle, posterior view. TM, Teres major; Lath, Lateral head of triceps; Loht, Long head of triceps; White arrows: posteromedial dividing bundle, black arrows: radial trunkasterix: radial nerve

The radial nerve systematically produced a minimum of four constant motor branches between the distal edge of the teres major muscle and the radial sulcus, one for the long head, one for the lateral head, two for the medial head, and then two sensory branches. A specific macroscopic appearance was found in 11 dissections: below the emergence of the branch of the long head of the triceps brachialis, the branches had a common emergence with the trunk of the radial nerve, giving the appearance of a posteromedial secondary common nerve trunk. (Fig. 4).

## Motor branch of the long head of the triceps

This is the first motor branch to emerge from the trunk of the radial nerve, apart from one case where it was the superomedial branch.

It was unique, except in one case, where two motor branches for the long head were found.

It emerged from the radial nerve at 1,3 ± 0,9 cm (min: -2,5, max :1, ratio 0,22), anterior to the distal edge of the teres major muscle, except in three cases, where the branch emerged at the distal edge of the teres major tendon, and in one case distally. It emerged on the posteromedial side of the radial nerve trunk, with an average diameter of 2.3 mm (min :1,5, max: 3). It ran medial to the radial nerve and crossed posteriorly over the medial brachial vein and ulnar nerve.

**Fig. 5:**
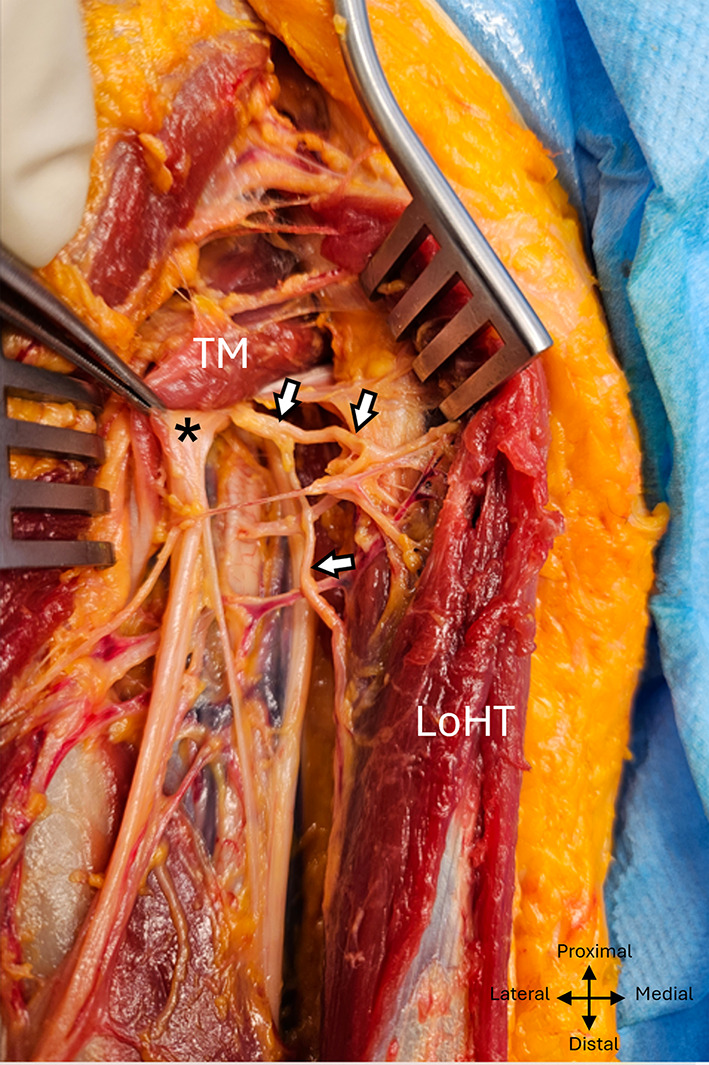
Branch of the long head of the triceps exposed posteriorly in the left arm. White arrows: branch of the long head. TM, Teres major; Loht, Long head of triceps; asterix: radial nerve

It divided into two at 0,5 ± 1 cm from the distal edge of the teres major muscle (min: -1, max: 3). (Fig. 5)

The first, transverse division reached the fleshy portion, proximal to the long head, under the proximal fascial lamina. Its diameter and length were 1,3 mm and 2,7 ± 1,1 cm respectively from the bifurcation, and it branched rapidly into three branches and, in nine cases, 4 branches.

The second division, oblique distally, crossed medially the vascular pedicle of the long head. It measured 4,5 cm ± 1,6 cm from its bifurcation with the transverse division, with a diameter of 1 mm, and in ten of the cases it showed early division in the first centimetre.

## Superomedial motor branch of the medial head

The second motor branch was the superomedial branch, destined for the medial and superior portion of the medial head.

It emerged from the radial nerve at 1,15 ± 1,6 cm from the inferior edge of the tendon of the teres major muscle (min: -4, max : 3,5, ratio : 0,30).

It then ran along the medial side of the radial nerve, heading medially towards the ulnar nerve. It divided into two at an average distance of 3.7 cm from its emergence. (min :0, max :8).

The first, long, medial division joined and ran alongside the ulnar nerve on its dorsolateral side. They both passed through the fibromuscular cleft formed by the convergence of the long and medial heads, meeting anteriorly and then on the medial side of the medial head. No exchange was found between the branch and the ulnar nerve. The point of penetration of the medial head of this division was located at an average length of 14,5 ± 3,6 cm (min: 6, max :21) from its emergence, i.e. 7.3 cm from the medial epicondyle. (min : 3 ; max :11,5).

**Fig. 6:**
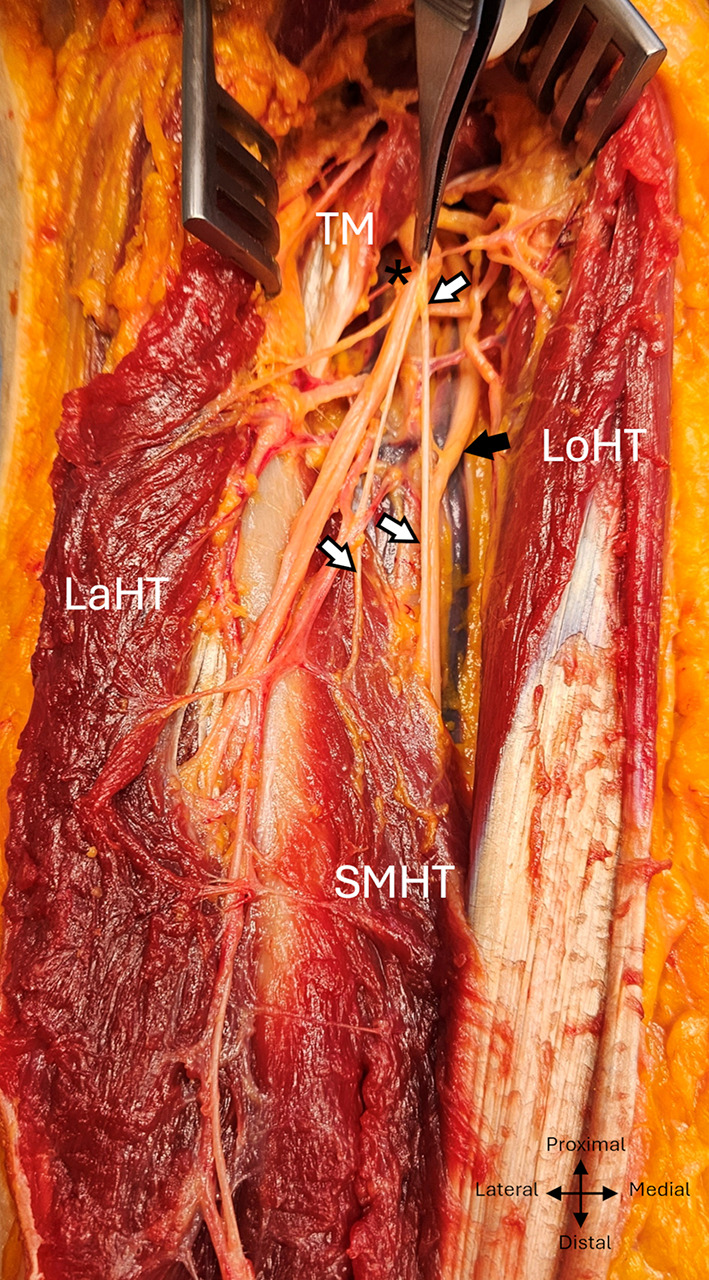
Superomedial branch exposed in the left arm via a posterior approach. White arrows: superomedial branch, black arrows: ulnar nerve. TM, Teres major; lath, Lateral head of triceps; loht, Long head of triceps; SMHT, Superior medial head of triceps; asterix: radial nerve

The second, vertical division had a more direct course, lateral to the first, medial to the radial nerve. It penetrated the superomedial portion of the medial head at its upper, superior and posterior end. It measured 7,5± 1,5 cm from its emergence. It was bifid in 12 of the 30 dissections. (Fig. 6)

### Motor branch of the lateral head

**Fig. 7:**
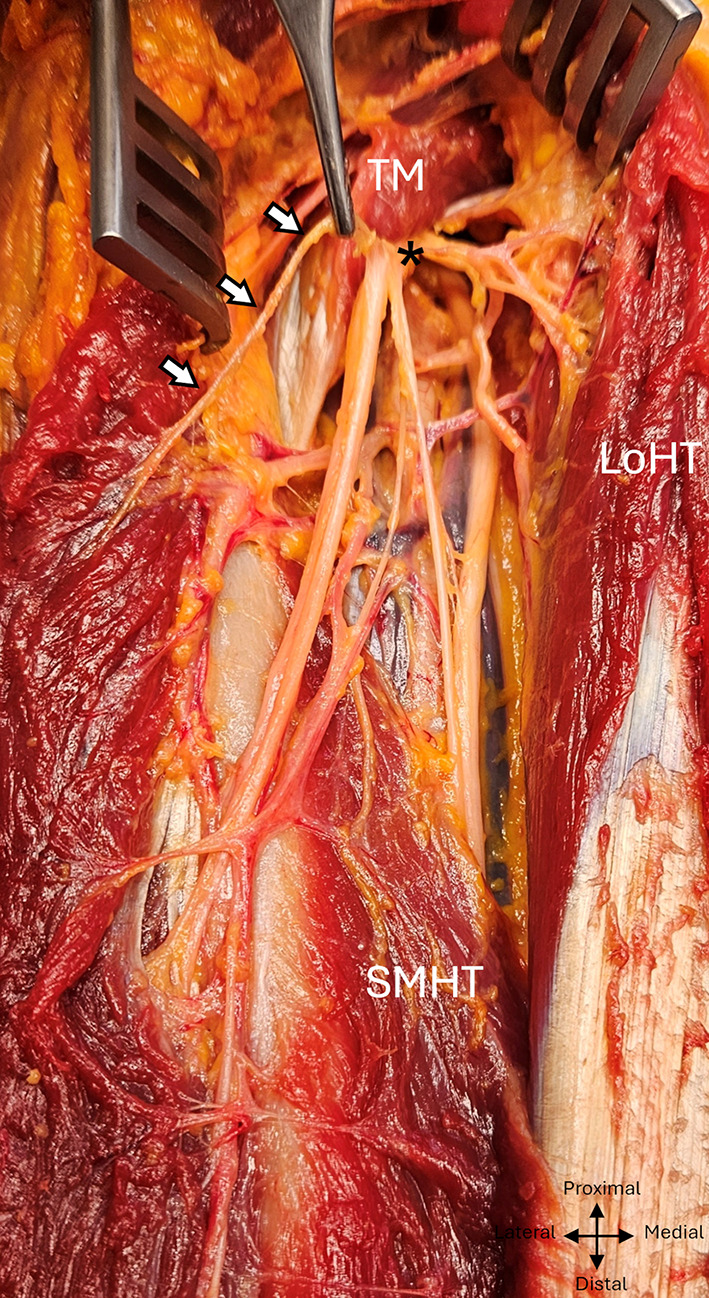
Branch of the lateral head, exposed to the left arm via a posterior approach White arrows: branch of the lateral head. TM, Teres major; Loht, Long head of triceps; SMHT, Superior medial head of triceps; asterix: radial nerve

It emerged on the lateral side of the radial nerve at 1,25 ± 1,3 cm (min: -0,5, max : 4, ratio : 0,30) from the lower edge of the tendon of the teres major muscle, with a diameter of 1.15 mm (min :0,5, max :2). Its lateral and distal oblique path had an average length of 4 ± 1,1 cm (min: 2, max :8). In half the cases, it divided into two at 1,4 ± 0,8 cm from emergence. The second branch of this division was 4,4 ± 1,1 cm long from emergence, its course being more oblique distally. In 4 cases, a second branch arose immediately from the radial nerve on its lateral side to the lateral head. The point of emergence was located at 3,5 ± 0,7 cm from the inferior edge of the tendon of the teres major muscle (min: 3, max: 4.5), then it headed distally and laterally, with a length of 2.8 cm. (Fig. 7)

## Inferomedial motor branch of the medial head

This is the last motor branch of the radial nerve for the triceps brachii and is destined for the inferior and deep portion of the medial head and anconeus muscle. It emerged on the medial side of the radial nerve at 3 ± 1,7 cm from the inferior edge of the tendon of the teres major muscle (min: -0,5, max: 6, ratio : 0,36), with an average diameter of 1.9 mm (mmin:1, max :3).

In 25 of the 30 dissections, branches emerged from it.

In 21 of the dissections, an additional branch for the lateral head emerged 3,4 ± 2,5 cm (min: 0, max: 8) from its length. This branch, 0.7 mm in diameter (min: 0.5, max: 1), ran laterally and obliquely distally for 3,7 ± 1 cm (min-max : 2–6), crossing behind the trunk of the radial nerve.

In nine dissections, a second branch originating from the inferomedial branch and destined for the lateral head emerged 5.1 ± 3.4 cm from its length, on its lateral side, with a lateral course, and oblique distally to the radial nerve, over a length of 3.3 ± 0.9 cm.

In one dissection, a third additional branch for the lateral head was found, emerging 10 cm from the inferomedial branch.

In ten dissections, the posterior cutaneous sensory branch of the forearm emerged from a terminal division of the inferomedial branch; of these, four had an additional branch for the lateral head and two had two.

**Fig. 8:**
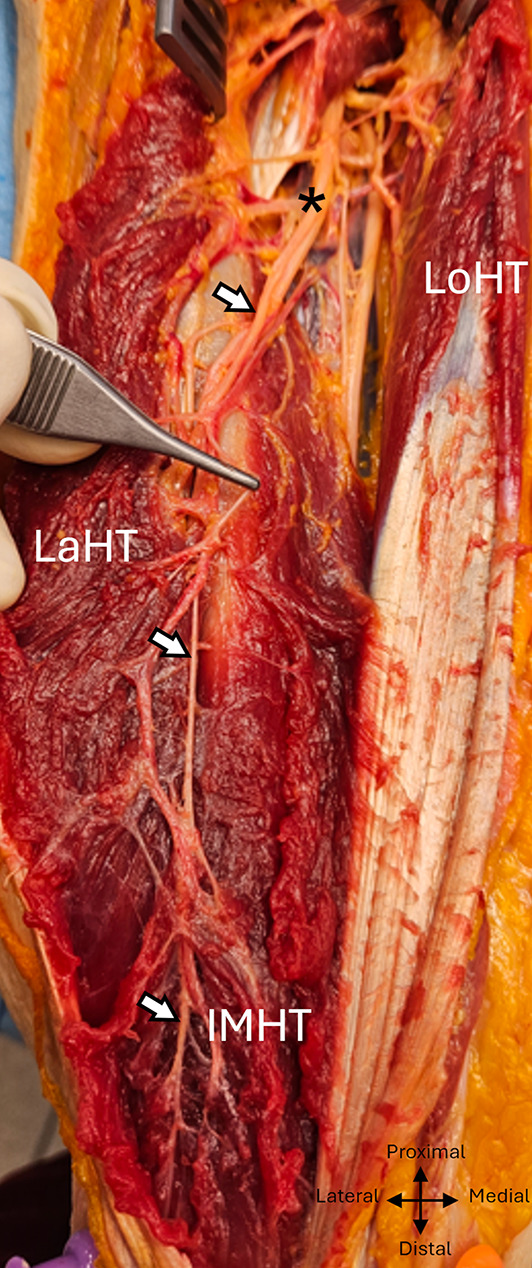
Inferomedial branch exposed posteriorly to the left arm. White arrows: inferomedial branch. Lath, Lateral head of triceps; Loht, Long head of triceps; IMHT, Inferior medial head of triceps; AM, asterix: radial nerve

**Fig. 9:**
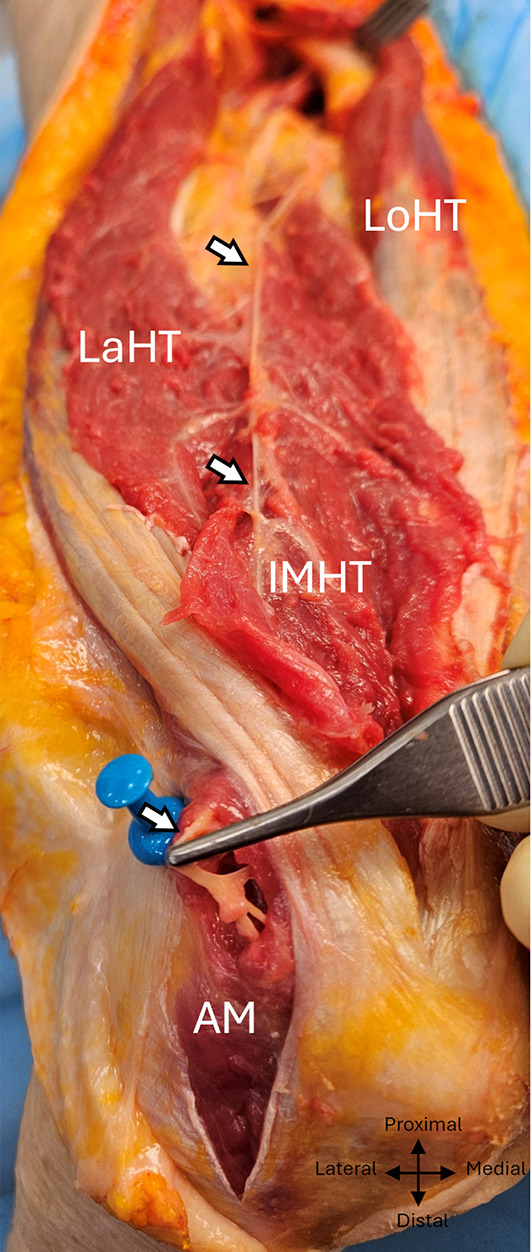
Inferomedial branch with its intramuscular course to the anconeus, exposed posteriorly in the left arm. White arrows: inferomedial branch, AM: anconeus muscle. Lath, Lateral head of triceps; Loht, Long head of triceps; IMHT, Inferior medial head of triceps; AM, Anconeus muscle.

The inferomedial branch to the medial deep head and anconeus had a post-division diameter of 1.1 mm and measured 19,4 ± 3 cm (min :12, max :24,5), including 6.8 ± 1.9 cm in the intramuscular pathway. It followed the radial nerve infero-medially in the radial sulcus, then, 11.5 ± 1.1 cm from the bi-epicondylar line, reached the superficial surface of the lower portion of the medial head, which it penetrated distally and laterally 6.8 ± 1.9 cm from this same line, thus becoming intramuscular. (min :4, max: 11). (Figures 8 and 9 et 10).

**Fig. 10:**
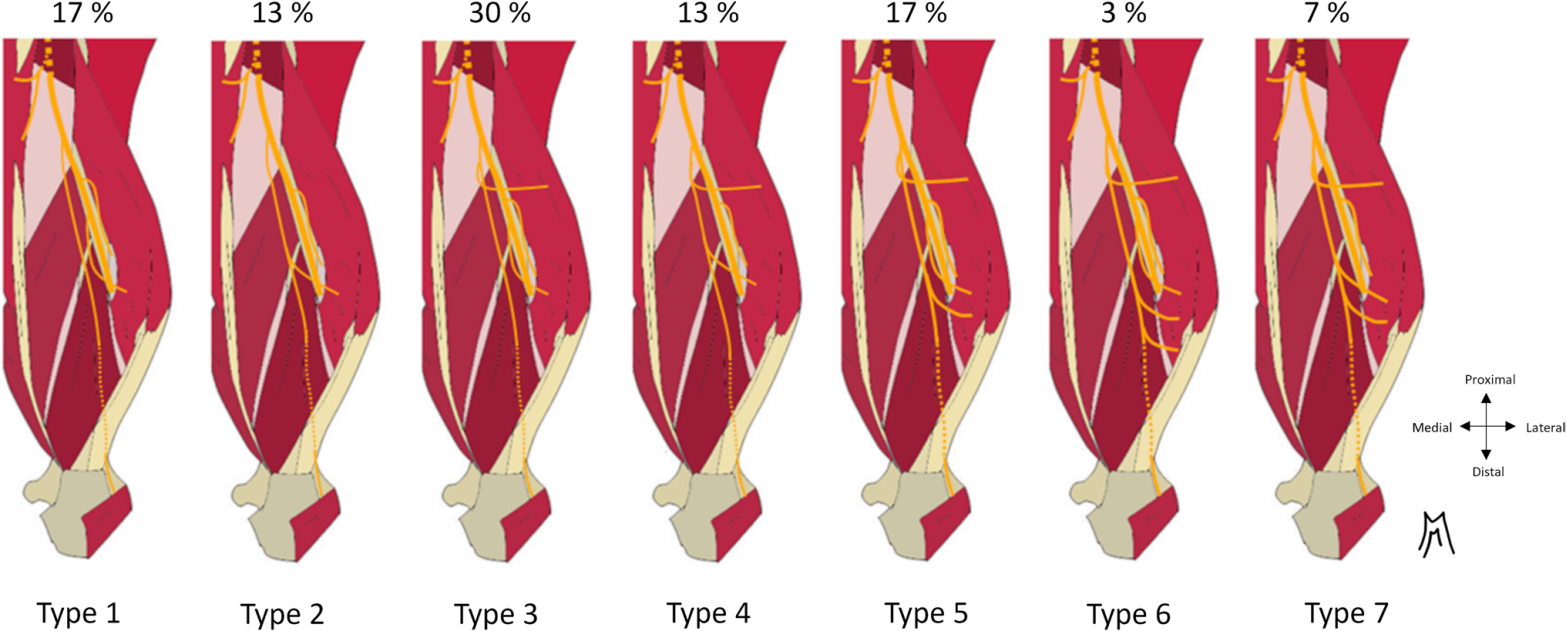
Schematic representation of the variants of the inferomedial branch according to the presence of additional branches.

## Discussion

The pattern of innervation of the triceps brachii by the radial nerve remains controversial and sometimes complex [16].

Several studies have suggested that the axillary nerve participates in the innervation of the triceps brachii, giving the branch of the long head of the triceps brachii [13, 14, 17]. 

Our study consistently found four distinct motor innervation branches of the radial nerve for the triceps brachii, one for the long head, one for the lateral head and two for the medial head. No involvement of the axillary nerve in triceps innervation was found. Our results are consistent with those of previous studies [8, 10–12].

However, we did not systematically find a second branch for the lateral head, which was present in only 25 of our 30 dissections, and which could come from the trunk of the radial nerve or, more often, from the inferior branch of the medial head. This branch, usually called the inferior branch of the lateral head, was found in a study of 18 of the 20 dissections [12]. We also found the existence of a second and, uniquely, a third branch for the lateral head arising from the inferior medial branch.

In other studies, the inferomedial branch could give rise to the posterior cutaneous sensory branch of the forearm or branches destined for the lateral head [6, 9, 11].

Taking the inferior branch of the medial head would most often require neurolysis to spare the branches destined for the lateral head arising from it and from the posterior cutaneous branch of the forearm. If the branch is taken proximally, there is a risk of transferring a sensory rather than a purely motor contingent.

In our study, the medial branch of the superomedial branch systematically accompanied the ulnar nerve. However, no communication was found between the two, in line with the results of other studies [18]. 

In more than a third of our dissections, we found a high division of the radial nerve branches into a common bundle distal to the branch of the long head, in agreement with the results of another study [12]. 

It should be noted that the radial nerve presents a fascicular aspect on the posterior surface of the humerus, and is easily dissected, which may contribute to the anatomical variations described.

Our study provides information on the constant arrangement of branches and potential variations. We also expressed in ratio the emergence of branches and structures improving inter-individual reproducibility.

Nevertheless, our study does not allow us to conclude on the functional axonal potential of the branches.

Furthermore, it would also be interesting to study the functional loss associated with denervation of one of the triceps brachialis heads, whether in its role as an extensor and stabiliser of the shoulder for the long head, or in its role as an extensor of the elbow for each of the muscle heads.

## Conclusion

The present study supports the fact that it appears simpler to harvest the branch of the long head of the triceps, as it is easily identifiable, of satisfactory calibre, purely motor and can be performed with a more limited surgical approach.

However, further functional studies evaluating the consequences of denervation of the long head of the triceps brachialis would be necessary to confirm that its motor branch can be safely harvested without significantly impacting shoulder stability.

## Data Availability

No datasets were generated or analysed during the current study.
